# Trends for Influenza-related Deaths during Pandemic and Epidemic Seasons, Italy, 1969–2001

**DOI:** 10.3201/eid1305.061309

**Published:** 2007-05

**Authors:** Caterina Rizzo, Antonino Bella, Cécile Viboud, Lone Simonsen, Mark A. Miller, Maria Cristina Rota, Stefania Salmaso, Marta Luisa Ciofi degli Atti

**Affiliations:** * Istituto Superiore di Sanità, Rome, Italy; † University of Bari, Bari, Italy; ‡ National Institutes of Health, Bethesda, Maryland, USA

**Keywords:** pandemic season, geographic trend, influenza, excess mortality, research

## Abstract

Age-specific patterns of death from influenza vary, depending on whether the influenza season is epidemic or pandemic. We assessed age patterns and geographic trends in monthly influenza-related deaths in Italy from 1969 through 2001, focusing on differences between epidemic and pandemic seasons. We evaluated age-standardized excess deaths from pneumonia and influenza and from all causes, using a modified version of a cyclical Serfling model. Excess deaths were highest for elderly persons in all seasons except the influenza A (H3N2) pandemic season (1969–70), when rates were greater for younger persons, confirming a shift toward death of younger persons during pandemic seasons. When comparing northern, central, and southern Italy, we found a high level of synchrony in the amplitude of peaks of influenza-related deaths.

## Abstract 2

During epidemics, excess deaths were similar in amplitude and time across 3 regions.

In Europe and the United States, the geographic pattern of influenza epidemics has been studied extensively, yet mostly at the national level with few local studies (*1**–**6*). Description of local influenza patterns can contribute to understanding of transmission and seasonality, which are influenced by factors such as demographic differences, climatic variability, and virus virulence.

Age patterns and geographic trends for influenza are commonly assessed by using data on influenza-related deaths, which are indirectly quantified by using statistical methods to estimate seasonal increases in death from pneumonia and influenza (P&I) or all causes (AC) (*7**–**11*). This approach has shown that age-specific influenza death patterns vary according to whether the influenza season is epidemic or pandemic. During epidemic seasons, proportion of influenza-related deaths is greatest among persons ≥65 years of age, whereas during all 3 influenza A pandemics in the 20th century, persons in this age group accounted for a lower proportion of influenza-related deaths in the United States (*12*) and Europe (*8**,**13*).

Although patterns of influenza-related deaths have been investigated in many countries (*14**–**18*), few studies have focused on southern Europe. With regard to Italy, these methods have been applied only to death data for elderly persons during 1970–2001 and only at the national level (*19*). Our objective was to use the above-described approach to assess age patterns and geographic trends for influenza-related deaths in Italy; our focus was on differences between epidemic and pandemic seasons.

## Methods

### Death and Population Data

We obtained the monthly number of deaths caused by P&I and AC in Italy from 1969 (first available data year) through 2001 (most recent data year) from the Italian National Census Bureau, which records all causes of death reported on death certificates and classifies them according to the International Classification of Diseases (ICD). For our analysis, we considered only the underlying cause of death. To select P&I deaths, we used ICD-8 codes 480–486 and 470–474 for the years 1969–1979 and ICD-9 codes 480–486 and 487 for the years 1980–2001.

The geographic areas considered were the 3 administrative regions of Italy: northern Italy, central Italy, and southern Italy, as defined by the Italian National Census Bureau (*20*). Northern Italy comprises Piedmont, Lombardy, Autonomous Province of Trento, Autonomous Province of Bolzano, Val d’Aosta, Veneto, Friuli Venezia Giulia, Liguria, and Emilia Romagna. Central Italy comprises Tuscany, Umbria, Marche, and Lazio. Southern Italy comprises Abruzzo, Molise, Campania, Puglia, Basilicata, Calabria, Sicily, and Sardinia. For each year, we generated summary datasets of the monthly number of deaths from P&I and AC, stratified by age group (0–14, 15–44, 45–64, and >65 years) (*9*). We calculated the annual number of persons in each age group and the monthly number of deaths per 100,000 population for each age group and standardized these to 30.4-day months.

### Virologic Surveillance

To determine which influenza viruses were circulating each season, we reviewed publications listing viral subtypes identified in Italian laboratories (*21**–**23*). For the most recent years (1999–2001), we obtained these data from the Italian National Influenza Center, which has performed virologic surveillance since 1999.

### Statistical Analyses

To estimate age-specific excess deaths an indirect measure of death attributable to influenza from P&I and AC for the 32 influenza seasons, we applied a Serfling-type regression model to monthly time series of deaths (*7**,**9*). As described in previous studies (*9**,**19*), we removed the seasonal trend from the time-series data (de-trended) by fitting a smooth spline function to the average death rates in summer (June–August). Then, we applied a seasonal regression model to the de-trended series, excluding values for December–April, to model the expected mortality rates in the absence of influenza activity. Monthly mortality rates were calculated as the observed minus the predicted mortality rates for all epidemic months. We identified epidemic months by applying the above-mentioned procedure to deaths coded specifically as influenza (ICD-8 code 470–474 and ICD-9 code 487). We defined epidemic months as those winter months for which influenza-specific mortality rates exceeded the upper 95% confidence limit of the seasonal model.

Seasonal excess deaths were estimated as the sum of monthly excess deaths, after back-adjusting for the true month length and removing the spline transformation. The model was applied to P&I and AC mortality rates separately for each age group. We achieved an excellent fit for all age groups. All model terms included were statistically significant (p<0.0001), but additional terms were not (p>0.05).

### Age Patterns and Geographic Trends

To determine whether variations in age structure biased the geographic comparisons, we generated excess mortality rates for each area and age group and standardized them on the basis of the age distribution of the Italian population in 2001 (the year of the most recent Italian census). This permitted a comparison of age-adjusted P&I and AC excess mortality rate estimates across areas.

To compensate for nondemographic differences among areas (e.g., differences in access to healthcare and in coding for cause-specific deaths) (*24*), we also calculated the percentage increase in mortality rates as the excess deaths divided by the baseline deaths in winter (expected deaths), for P&I and AC, separately. This measure has been used successfully in past research (*7**,**8*). To estimate correlations of influenza-related death across the 3 geographic regions, we calculated the pairwise Spearman correlation coefficients of seasonal estimates for the 32 years considered.

## Results

### Geographic Trends, Synchrony, and Effect of Epidemic Seasons (All Ages)

For the 32 influenza seasons studied, excess deaths averaged 3 per 100,000 population (range 0–38) for P&I and 18 per 100,000 for AC (range 0–107). Influenza accounted for an estimated 57,243 deaths from P&I (average 1,789 per season) and 318,806 from AC (average 9,963 per season).

**Figure 1:**
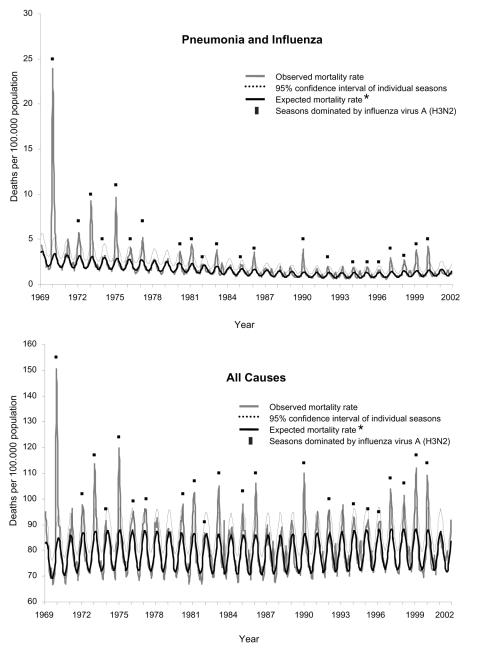
Monthly mortality rates from pneumonia and influenza and all causes for Italy, January 1969–December 2001. *Baseline mortality rates determined by Serfling model.

The highest number of excess deaths was found for the 1969–70 pandemic season; no measurable number of excess deaths was found for 5 seasons (1981–82, 1984–85, 1986–87, 1990–91, 2000–01) (Figure 1). The 27 seasons that had excess P&I and AC deaths had an average of 2.4 epidemic months per season (range 1–4). The influenza seasons with higher excess deaths tended to be characterized by a predominance of influenza A (H3N2) viruses (Figure 1). For these seasons, the average excess deaths from P&I and AC (4.5 and 23.4 per 100,000 population, respectively) was 4× higher than that for the 11 seasons in which influenza A (H1N1) or B viruses were predominant (0.8 for P&I and 7.4 for AC, per 100,000 population).

**Figure 2:**
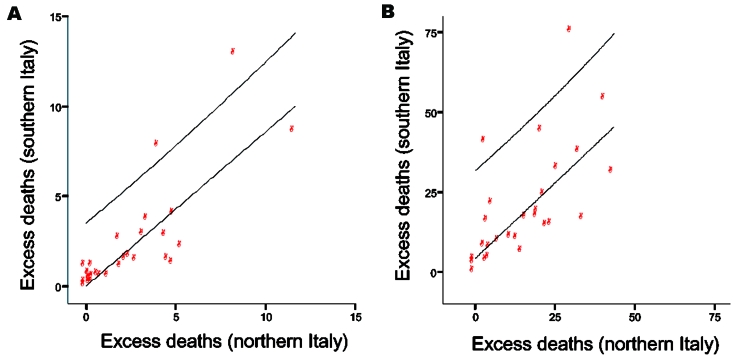
Correlation in the influenza epidemics for 31 influenza seasons (1970–2001), measured by excess deaths from pneumonia and influenza (A) and all causes (B). Excess mortality rates per 100,000 in southern and northern Italy.

For the overall study period, the excess deaths per 100,000 population from AC was 15 for northern Italy, 14 for central Italy, and 22 for southern Italy; from P&I they were 4, 3, and 3, respectively (Appendix Figure 1). Also for these 32 years, no statistical differences among the 3 geographic areas were noted for excess deaths from P&I or AC (Kolmogorov-Smirnov test, p = 0.8 and p = 0.9, respectively). Patterns were similar with the percent increase in excess deaths from P&I and AC. The 95% confidence intervals for estimates for individual seasons were within 6% of given values (Table 1). When conducting this analysis for seasons in which influenza A (H3N2), A (H1N1), and B predominated, area-level differences were again not significant. The strong correlation of excess-death estimates in the 3 regions suggests a high level of synchrony in the amplitude of local influenza epidemics (number of excess deaths peaked in the same month in each region) across Italy (P&I, Spearman ρ = 0.88–0.93, p<0.0001; AC, Spearman ρ = 0.80–0.93, p<0.0001) (Figure 2; Appendix Figure 2).

**Table 1:** Mean age-standardized excess all-cause deaths per 100,000 population and percent increase in death rates and the winter seasonal percent increase attributable to influenza, Italy, 1969–2001*

Deaths	Northern	Central	Southern
Pandemic season (1969–1970)			
Excess	103.6	85.3	105.2
Percent increase	21.7	21.0	25.9
Epidemic seasons (1970–2001)			
Excess	13.7	13.2	17.1
Percent increase	3.4	3.6	5.1
Entire study period (1969–2001)			
Excess	16.6	15.4	19.9
Percent increase	4.0	4.1	5.7

*95% confidence intervals for individual season estimates were within 6% of values listed.

### Magnitude and Trends of Influenza-related Deaths during Pandemic and Epidemic Seasons, by Age

During epidemic seasons, most influenza-related deaths at the national level (84%) occurred in persons ≥65 years of age, for P&I and AC; by contrast, during the 1969–70 influenza A (H3N2) pandemic season, deaths markedly affected all age groups, especially the 45–64 group.

In Italy the proportion of excess deaths from AC in persons <65 years of age was 3-fold higher during the pandemic season than during all other epidemic seasons. In particular, when the pandemic season was compared with the season with the second highest number of deaths (1974–75), the number of influenza-related deaths was 7× higher for persons 0–14, 4× higher for persons 15–44 and 45–64, and 2× higher for persons ≥65 years of age. Similar results were obtained for all 3 geographic areas (Table 2). The number of excess deaths from AC during the influenza A (H3N2) pandemic season was 1- to 9-fold higher in Italy than in other European countries (France, England, and Wales), in North American countries (United States, Canada), and in Asian countries (Japan, Australia) (Table 3).

**Table 2:** Mean age-standardized excess deaths per 100,000 population, Italy, 1969–2001

Age, y	Pneumonia and influenza						All causes					
	1969–70			1970–2001			1969–70			1970–2001		
	Northern	Central	Southern	Northern	Central	Southern	Northern	Central	Southern	Northern	Central	Southern
0–14	2.4	3.3	7.8	0.0	0.1	0.1	8.6	8.7	30.3	0.3	0.6	1.6
15–44	3.8	2.5	3.0	0.1	0.1	0.1	7.7	7.0	8.4	0.6	0.8	0.8
45–64	38.8	25.4	27.4	0.7	0.4	0.7	112.1	75.1	109.0	4.3	4.3	6.6
≥65	288.7	221.8	218.2	14.0	12.7	14.2	694.8	621.6	859.2	75.6	71.2	115.7
Total	43.0	31.5	29.3	2.2	1.9	1.9	103.6	85.3	105.2	12.0	11.6	18.6

**Table 3:** Multinational comparison of influenza A (H3N2) Hong Kong pandemic, based on all-cause excess deaths estimates*

Deaths	Italy†	England, Wales†	France†	Australia‡	Japan†	USA§	Canada§
Overall no./100,000 population	107	77	72	64	49	27	12
Increases over baseline, %	24	20	21	16	20	8	6
Persons <65 y,	29	23	27	20	N/A	34	24

*Data from this study and (*8*). Estimates are for the major pandemic seasons, for which timing varied by country. N/A, not applicable. †Second season of virus circulation, 1969–70. ‡Second season of virus circulation, 1970. §First season of virus circulation, 1968–69.

## Discussion

This study showed a high level of correlation in the amplitude of influenza epidemics (i.e., peaks in rates were similar) in the 3 Italian regions during a 32-year period spanning epidemic and pandemic seasons. The analysis of local influenza-related death patterns did not show differences in mean mortality rates among geographic areas These findings are consistent with the high level of synchrony found in other area-level studies in Europe and in the United States (*1**,**2**,**5**,**6*).

The first season analyzed was the 1969–70 pandemic season. In Italy, as in other European countries (*8*), the pandemic season was more destructive in the second season of circulation of influenza A (H3N2) virus (i.e., in 1969–70), 1 year after the pandemic strain was first introduced to Italy (*25**–**27*). The pandemic season seems to have had a greater effect in Italy; excess mortality rates were estimated to be 38 (20,000 deaths) for P&I and 107 (57,000 deaths) for AC. These unexpectedly large excess mortality rates were 3-fold higher than that in the United States and 1-fold higher than those in other European countries. The increase in percentage of deaths reduced but did not eliminate these differences. However, the percentage of deaths in persons <65 in Italy (29%) was lower than the percentage in that age group in the United States (34%) but similar to the percentages in other European countries, especially France (27%) (Table 3). Future studies could address these differences in numbers of deaths that may stem from underlying differences in baseline mortality rates or perhaps in influenza transmission.

During the pandemic seasons, compared with epidemic seasons, the relative increase in mortality rates was lower for elderly than for younger persons in Italy, confirming that during pandemics, children and young adults have a greater relative risk for death than the elderly (*12*). A possible explanation is the partial immune protection of elderly persons who may have been exposed before 1891 to H3 antigens (*28*), whereas persons born after 1891 would not have been exposed to these antigens.

Several limitations should be mentioned. First, deaths from P&I were not always confirmed by laboratory methods, which could have resulted in misclassification of deaths. However, patterns of death from P&I were very similar to those from AC, which are not subject to this bias. A second limitation was that demographic and nondemographic differences could have biased the geographic comparison. However, we performed age standardization and calculated the percentage increase in deaths over the winter baseline, which reduces baseline differences in deaths, unrelated to influenza.

A third, more critical, caveat stems from the surveillance of viral subtypes. The proportion of laboratory-confirmed cases has only been available since 1999 (i.e., for only 2 years of the study period). However, in the latest years the proportion of laboratory-confirmed cases was ≈15% (range 11%–28%), with >3,000 samples tested (*29*), which could have affected the accuracy of influenza diagnoses over time and perhaps across regions. For example, during the 1998–99 season, when influenza B viruses were predominant, the death rate was high compared with that found for the other influenza B seasons, which indicates that the characterization of viral subtypes is limited by the geographic distribution of the sites participating in virologic surveillance.

Our findings suggest that influenza epidemics are strongly correlated in amplitude across the 3 regions of Italy. Different factors have been reported to drive the spatial and temporal correlations of epidemics: population movements and environmental factors such as climate or weather conditions (*5**,**30**,**31*). Although population movements are assumed to play a key role in the global spread of influenza epidemics, they have been quantified only in the United States (*5**,**32**,**33*). The role of environmental factors and differences in circulating viruses among the geographic areas in Italy also remains to be clarified.

In conclusion, our results suggest that geographic synchrony of influenza in Italy is high and that for persons <65 years of age, death rates are likely to be substantially elevated in a future pandemic as compared with other epidemic seasons. Our study adds to others that have found strong spatiotemporal patterns in illness and death from influenza in the United States, France, Australia, and across Europe (*1**,**3**,**5**,**34*). Such results provide insight for the Italian pandemic preparedness and response efforts (*35**,**36*) and could be used in mathematical models for influenza spread at the national level.

## Supplementary Material

Appendix Figure 1 Correlation in the influenza epidemics for 31 influenza seasons (1970-2001), measured by excess mortality rates for pneumonia and influenza (panel A) and all causes (panel B) for 3 areas of Italy.

Appendix Figure 2 Monthly mortality rates from pneumonia and influenza and all causes for 3 areas of Italy, January 1969-December 2001. *Baseline of expected mortality rates determined by Serfling model.
