# Supplementary figures and images for: Trends for Influenza-related Deaths during Pandemic and Epidemic Seasons, Italy, 1969–2001
Source: Emerg Infect Dis. 2007 May;13(5):694–9. doi: 10.3201/eid1305.061309 (PMC2738436; doi:10.3201/eid1305.061309)

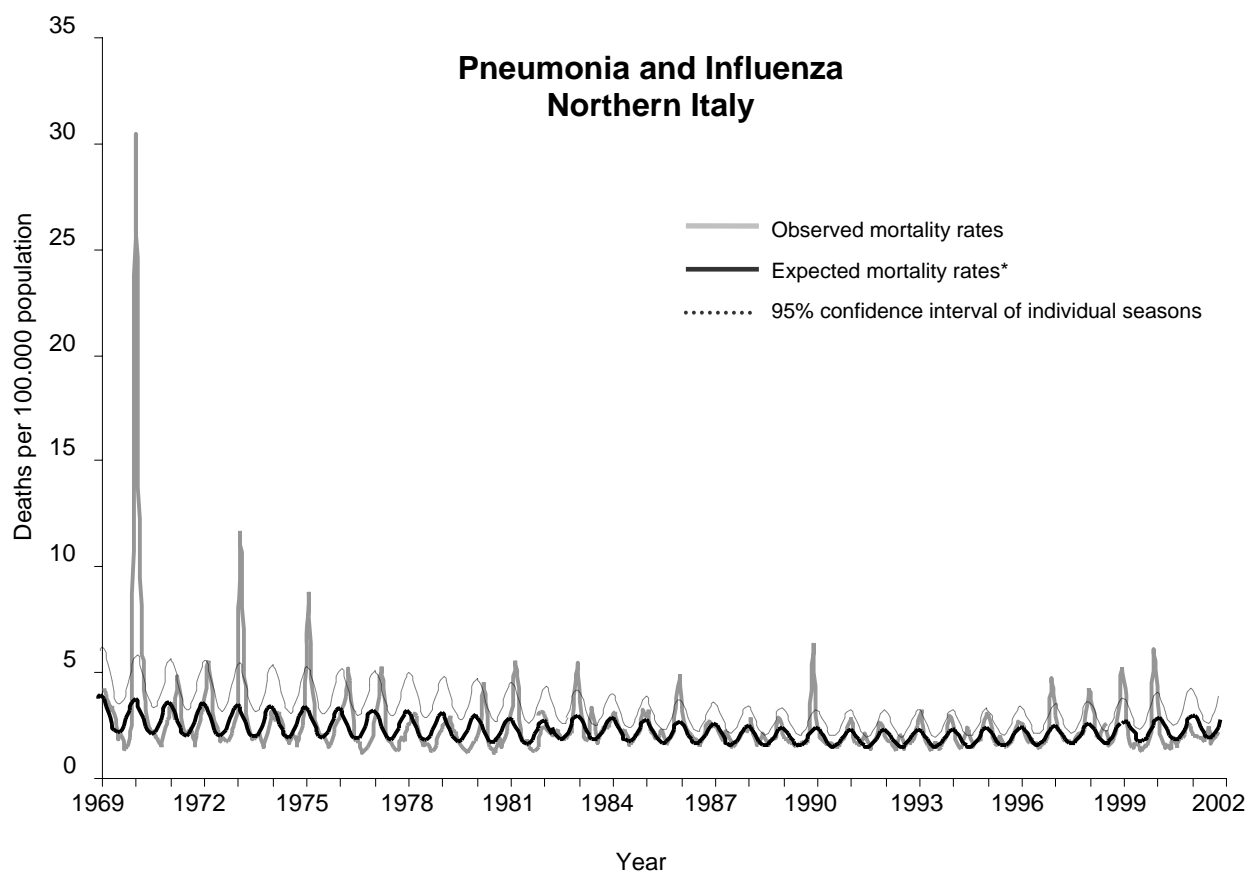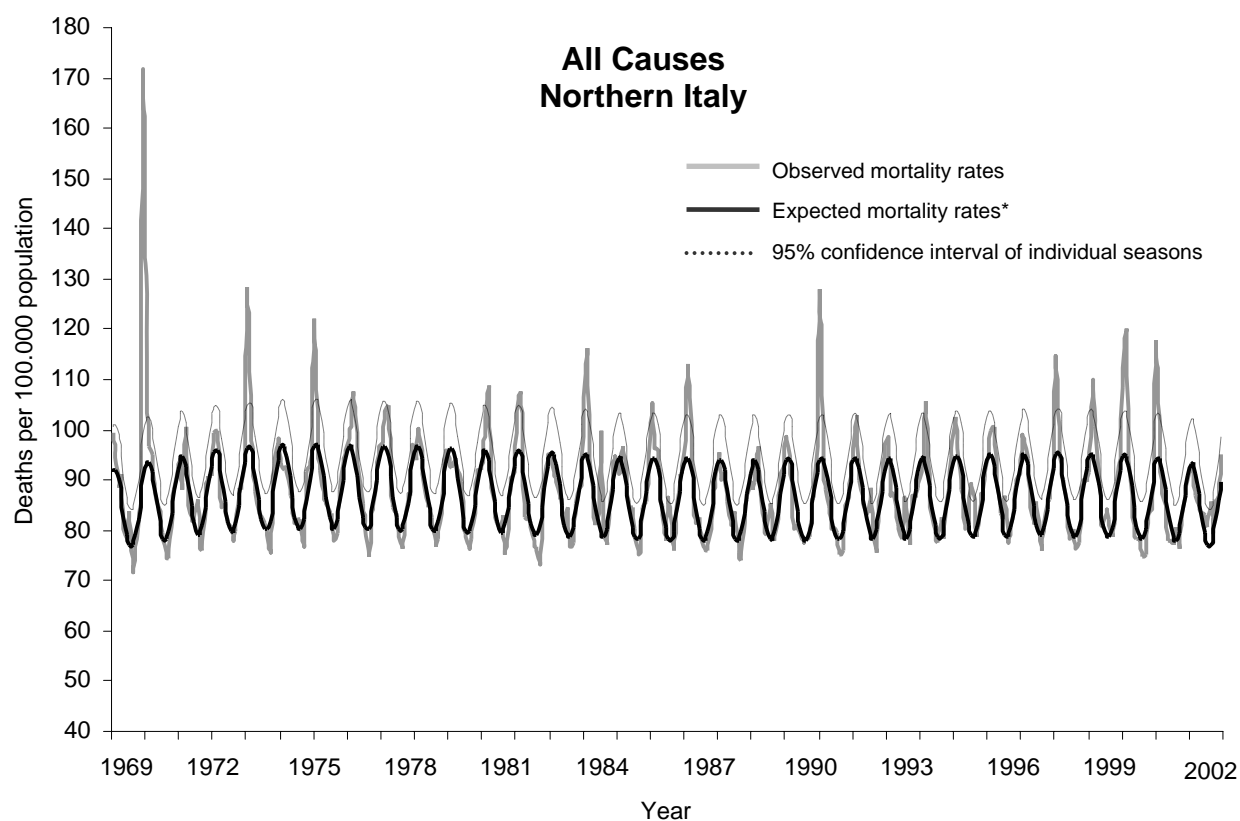

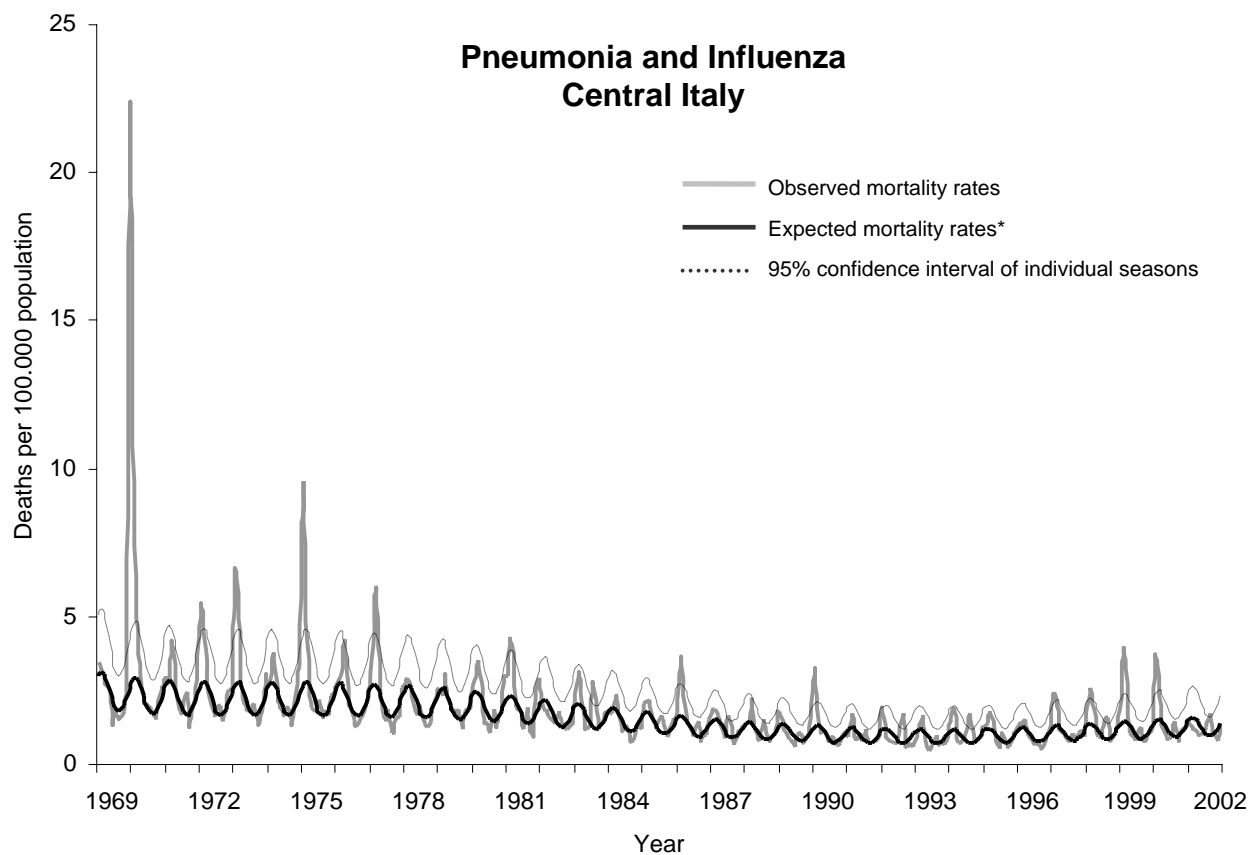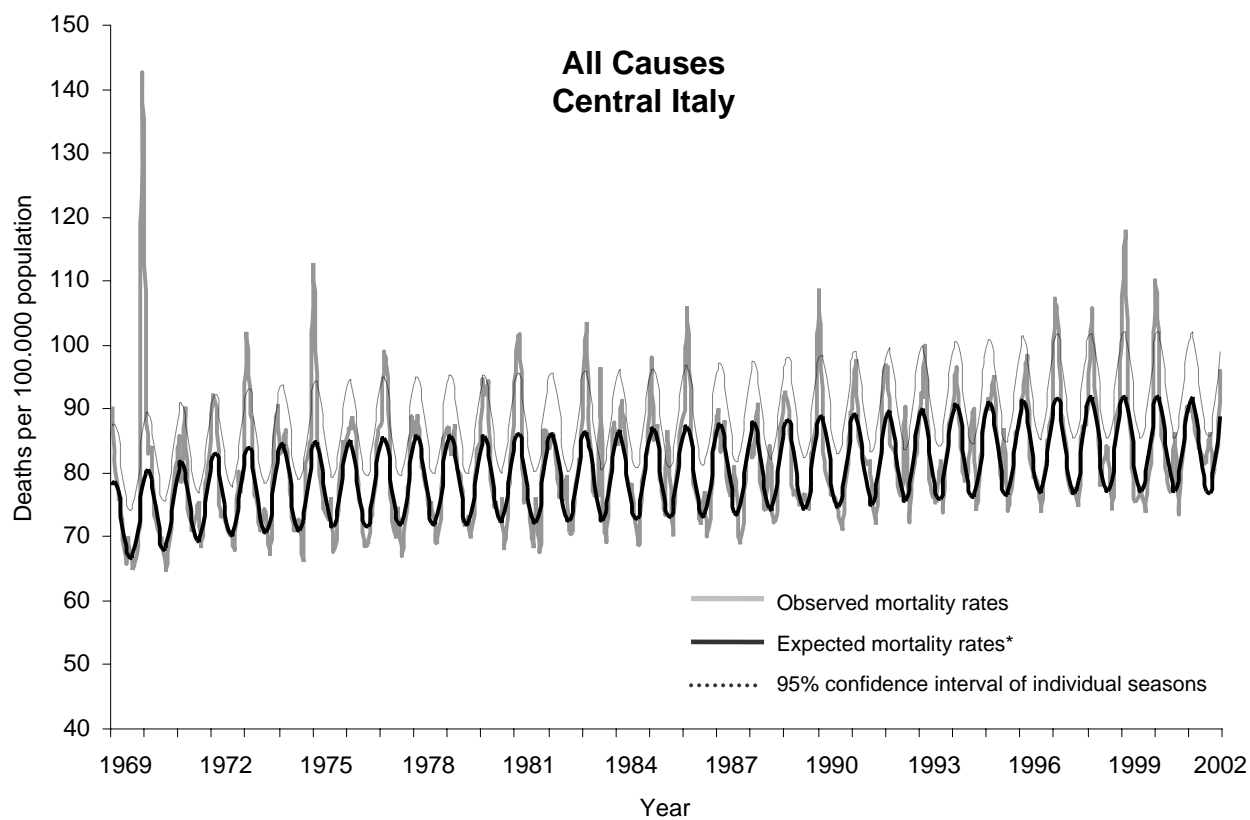

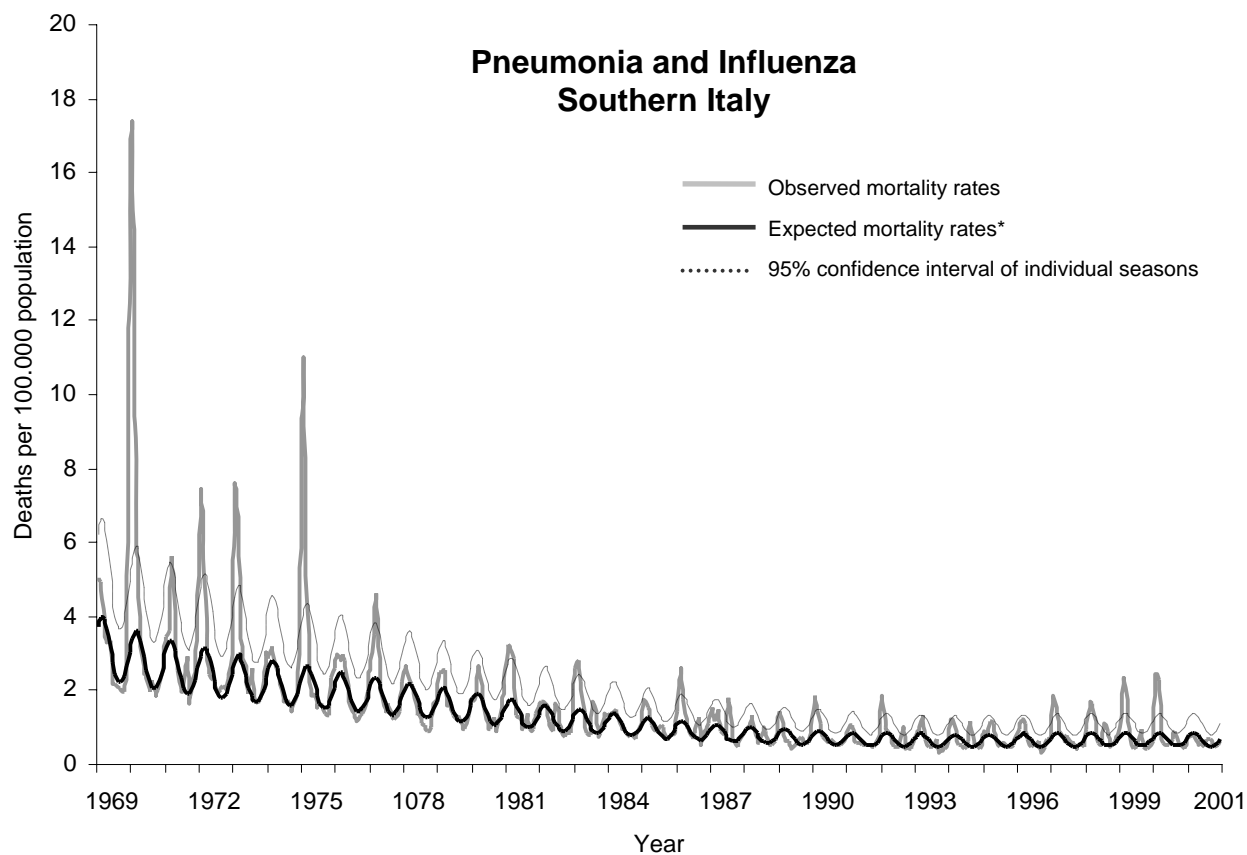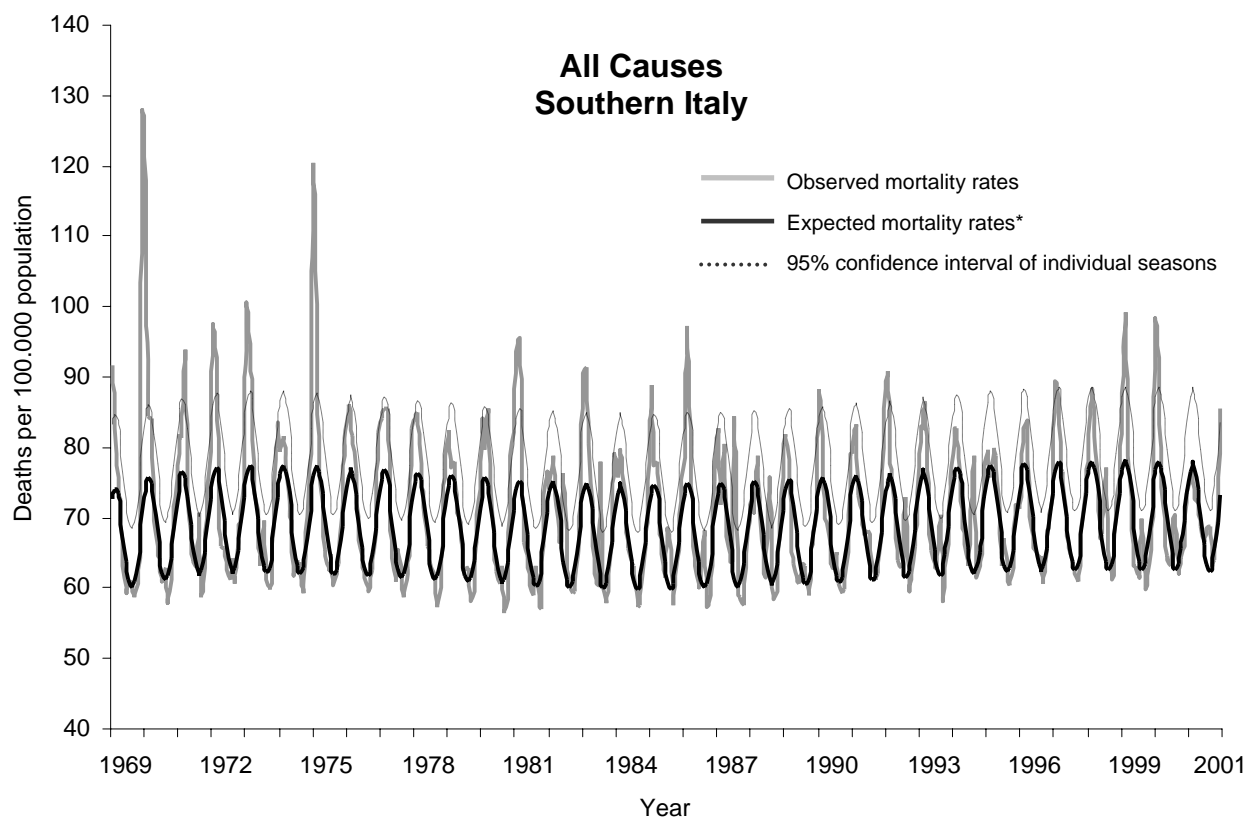

Supplement: Appendix Figure 1 — Correlation in the influenza epidemics for 31 influenza seasons (1970-2001), measured by excess mortality rates for pneumonia and influenza (panel A) and all causes (panel B) for 3 areas of Italy. [file 06-1309_appF1-s1.pdf]

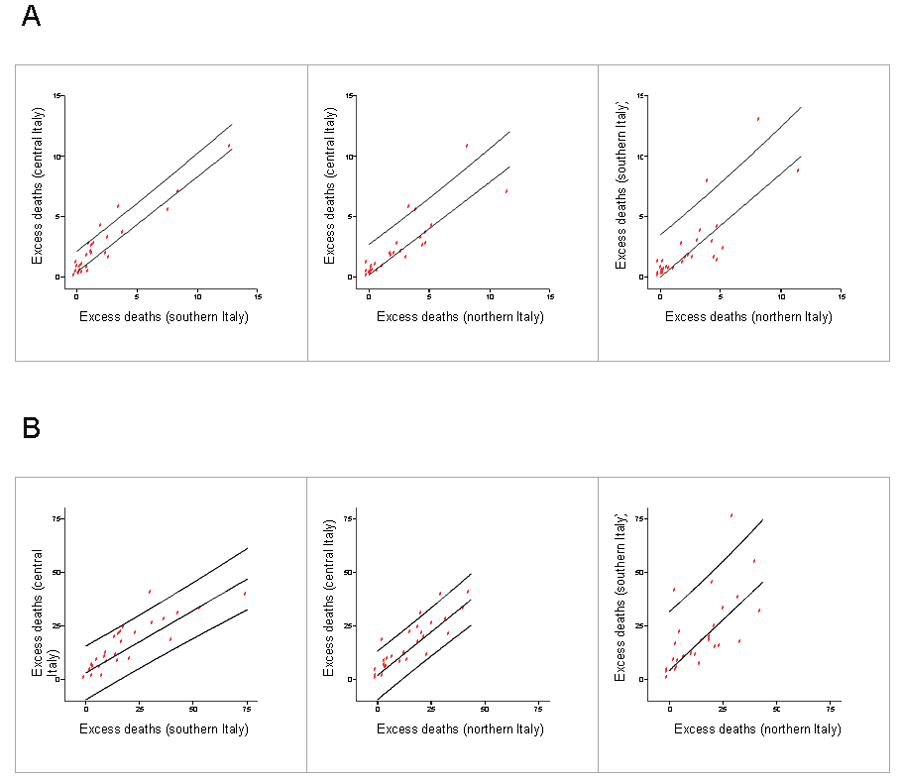

Supplement: Appendix Figure 2 — Monthly mortality rates from pneumonia and influenza and all causes for 3 areas of Italy, January 1969-December 2001. *Baseline of expected mortality rates determined by Serfling model. [file 06-1309_appF2-s2.gif]
